# A Statistical Method for Estimating Activity Uncertainty Parameters to Improve Project Forecasting

**DOI:** 10.3390/e21100952

**Published:** 2019-09-28

**Authors:** Mario Vanhoucke, Jordy Batselier

**Affiliations:** Faculty of Economics and Business Administration, Ghent University, Tweekerkenstraat 2, 9000 Gent, Belgium; jordy.batselier@ugent.be

**Keywords:** project management, entropy, managerial effort, distribution fitting, lognormal distribution

## Abstract

Just like any physical system, projects have entropy that must be managed by spending energy. The entropy is the project’s tendency to move to a state of disorder (schedule delays, cost overruns), and the energy process is an inherent part of any project management methodology. In order to manage the inherent uncertainty of these projects, accurate estimates (for durations, costs, resources, *…*) are crucial to make informed decisions. Without these estimates, managers have to fall back to their own intuition and experience, which are undoubtedly crucial for making decisions, but are are often subject to biases and hard to quantify. This paper builds further on two published calibration methods that aim to extract data from real projects and calibrate them to better estimate the parameters for the probability distributions of activity durations. Both methods rely on the lognormal distribution model to estimate uncertainty in activity durations and perform a sequence of statistical hypothesis tests that take the possible presence of two human biases into account. Based on these two existing methods, a new so-called statistical partitioning heuristic is presented that integrates the best elements of the two methods to further improve the accuracy of estimating the distribution of activity duration uncertainty. A computational experiment has been carried out on an empirical database of 83 empirical projects. The experiment shows that the new statistical partitioning method performs at least as good as, and often better than, the two existing calibration methods. The improvement will allow a better quantification of the activity duration uncertainty, which will eventually lead to a better prediction of the project schedule and more realistic expectations about the project outcomes. Consequently, the project manager will be able to better cope with the inherent uncertainty (entropy) of projects with a minimum managerial effort (energy).

## 1. Introduction

Project Management is the discipline to manage, monitor and control the uncertainty inherent to projects. Project management processes are used to monitor and control the progress of projects in order to reduce the uncertainty, and each such process requires effort from the project manager and her team. The academic literature has been overwhelmed by research studies in project management and control, and many of them focus on the construction of the project baseline schedule to assess the project risk and to monitor the performance of a project in progress. The combination of these three dimensions—schedule, risk and control—is often referred to in the literature as *dynamic scheduling* [1,2] or *integrated project management and control* [3].

This paper starts with the observation that the relation between managerial effort and the ability to reduce the project uncertainty lies at the heart of many research studies, although this relation is often not explicitly mentioned. Especially in some research papers that rely on the concept of *entropy* as a way to express that projects have the natural tendency to move to a state of disorder, authors have referred to the relation between entropy (uncertainty) and energy (effort). They have proposed different entropy measures to enable the project manager to better predict the project uncertainty and eventually reduce it by taking better actions. This concept of entropy is—to the best of the authors’ knowledge—not widely used in the previously mentioned dynamic scheduling studies; however, it is believed that it sheds an interesting light on the project management domain and opens ways to look at the dynamic scheduling literature (schedule/risk/control) in a fundamentally different way.

The current study reviews the research on entropy in project management and proposes a new way to accurately estimate project uncertainty to improve project forecasting and decision-making. This paper first elaborates on the link between the traditional dynamic scheduling literature and the much less investigated concept of entropy in project management and argues that entropy is an ideal concept to measure project uncertainty. Then, it will be shown that, in order to reduce a project’s entropy, forecasting and estimates are crucial for a project manager and her team to make well-informed decisions. Then, finally, a new so-called *calibration method* is proposed that should help project managers to better quantify the project uncertainty by providing better estimates for the activity durations. Such calibration procedures are relatively new in the literature, since they rely on a combination of statistical data analysis and the correction for human biases.

The paper is organized in the following sections. Section 2 reviews the most important studies on entropy for managing projects that have been used as an inspiration for the current research study. Based on this, the section also explains the basic idea of calibrating project data to better estimate project uncertainty, which constitutes the main theme of our study. In Section 3, two currently known data calibration methods from literature are then briefly reviewed, as they will be used as foundations for a new third calibration method taking the shortcomings of the existing methods into account. This new so-called *statistical partitioning heuristic* is discussed in Section 4. Section 5 presents the results of a computational experiment on a set of 83 empirical projects (mainly construction projects) from a known database. The section shows that the statistical partitioning heuristic outperforms the two other procedures, but also discusses some limitations that can be used as guidelines for future research. Finally, Section 6 draws conclusions and highlights some potentially promising future research avenues.

## 2. Managing Projects

### 2.1. Entropy in Project Management

Project Management is the discipline to manage, monitor and control the uncertainty inherent to projects. Whatever specific project management process is used to monitor and control the project progress to reduce the uncertainty, it always requires effort from the project manager and her team. In several studies in the literature, this managerial effort of project management to reduce the project’s uncertainty is studied from an entropy point-of-view. In this view, the entropy is the natural tendency of projects to move to a state of disorder, often quantified as schedule delays, cost overruns and/or quality problems, and the managerial effort to monitor and control such projects in progress is then the *energy* of the entropy concept to reduce the uncertainty. The general idea of entropy is proposed by [4] who stated that the uncertainty of a system decreases by receiving information about the possible outcome of the system. From this point of view, *project management* requires energy to cope with the inherent entropy of projects. Note that the term energy cannot be interpreted in a very strict sense here, since energy itself is of course not sufficient for dealing with entropy. Project management is much more than just using energy, and instead requires the right people at the right place to solve problems. Hence, effective project management requires “competences” and “skills” which are composed by many components, and not only the amount of energy by its people. Consequently, the term *energy* is used to refer to all the effort done by people with the right competences to bring projects in danger back on track.

Most project management studies do not explicitly take the concept of entropy into account, but nevertheless all aim at developing new methodologies for project managers to better measure, predict and control the inevitable problems of a project (uncertainty) in the easiest possible way (effort). Consequently, while many excellent studies indirectly deal with the issue of managing project uncertainty, to the best of our knowledge, only three studies explicitly quantified the relation between managerial effort (*energy*) and uncertainty reduction (*entropy*). First, the study of [5] investigated whether the use of *schedule risk analysis* can improve the time performance of projects in progress. In a large simulation study with artificial project data, the author varied the degree of management attention—which is a proxy for the effort of control—and measured whether this has an impact on the quality of the corrective action decision-making process to bring projects in trouble back on track (uncertainty reduction). The study of [6] extended this approach and relied on the same concept of effort (of a project manager) and quality of actions (to cope with uncertainty) and compared two alternative project control approaches. The bottom-up control approach is similar to the previously mentioned schedule risk analysis study and aims at reducing the project uncertainty by focusing on the activities with the highest risk in the project schedule. The second so-called top-down method makes use of the well-known earned value management methodology to monitor the project’s performance, which is used as an early warning signal for taking corrective actions. The authors compared these two alternative project control methods, and proposed the so-called *control efficiency* concept which aims at finding the right balance between minimizing effort and maximizing quality of actions. Finally, Ref. [7] measured the impact of managerial effort to reduce the activity variability on the project time and cost performance. Without mentioning the concept of entropy, they defined a so-called effort-uncertainty reduction function to quantify the relation between the managerial effort (energy) and the reduced uncertainty (entropy). Despite the explicit quantification of both *effort* and *uncertainty reduction*, these three studies never have made any attempt to use empirical project data to measure uncertainty. Instead, all results have been obtained using simulation studies on artificial project data using statistical probability distributions with randomly selected values for their parameters to quantify project uncertainty. Hence, since the authors had no idea whether the chosen values correspond with possible real-life values, they have relied on a huge set of simulation runs, varying these values as much as possible to assure that their results provide enough managerial insights relevant for practice. Moreover, none of these studies have explicitly referred to the concept of entropy as a possible way to model project uncertainty.

However, the use of entropy sheds an interesting light on the project management domain. In a study of two decades ago by [8], the authors proposed an entropy model for estimating and management the uncertainty of projects, and argued that controlling projects comes with a certain degree of managerial effort, since:

“With the aid of the entropy one can estimate the amount of *managerial effort* required to overcome the *uncertainty* of a particular project.”

Or course, not all project management studies took the relation between effort and uncertainty so explicitly into account, but nevertheless made use of the entropy concept in project management. Ref. [9] proposed an uncertainty index as a quantitative measure for evaluating the inherent uncertainty of a project, and analysed their approach on a real turbojet engine developing project. In a recent study, Ref. [10] measure the uncertainty related to the evolution of a resource-constrained project scheduling problem with uncertain activity durations using the entropy concept. Ref. [11] proposed a new risk analysis and project control methodology, and used entropy functions for a project’s completion time and critical path. In addition, [12] proposed an entropy-based approach for measuring project uncertainty, and argued that management’s inability to address uncertainty is one of the major reasons for project failures. According to these authors, the managerial effort to deal with uncertainty in projects should consist of three parts:

**Step 1.** Identifying sources of project uncertainty,
**Step 2.** Quantifying project uncertainty,
**Step 3.** Using the uncertainty metrics for improving decision-making.

The previously mentioned studies have been an inspiration to develop and propose the model of the current paper. However, it should be noted that the literature contains many studies dealing with the three-step process discussed earlier, and an overview of these is outside the scope of this paper. The reader is referred to summary papers about project risk [13] and project control [14] to find interesting references. The current study elaborates on the second part of the required managerial effort (*quantifying uncertainty*) and proposes a new way of quantifying probability distributions for activity duration by making use of empirical project data rather than simply by relying on statistical probability functions with randomly chosen values for the averages and variances (with no known link to practice). Ref. [8] argue that such a study for better quantifying activity duration uncertainty is necessary since “usually in practice we can only estimate the possible duration range of activities and very rarely we have information about the probability distribution curve”. Moreover, in the previously mentioned paper by [12], the authors conclude that “a better prediction of project costs, schedule and potential benefits leads to more realistic expectations about project outcomes and lower failures”, and, hence, implicitly argue that a more accurate way of estimating probability distributions for project uncertainty is key for making better project management decisions.

As a conclusion, the previous studies have shown that, just like any physical system, projects have entropy that must be managed by spending energy. This energy process—defined as all the effort done by people with the right competences—is a very important aspect of any project management methodology. In order to manage the inherent uncertainty of these projects, accurate estimates (for durations, costs, resources, …) are crucial to make informed decisions. Without these estimates, managers have to fall back to their own intuition and experience, which—although valuable—are often subject to biases and hard to quantify. The next section discusses the specific approach of the current study to accurately estimate distributions for activity duration, and it is shown that this specific approach—which we refer to as data calibration—is an extended version of an existing methodology of three recently published studies.

### 2.2. Calibrating Data

In the previous section, it has been shown that forecasting is important for good decision-making in project management, and that such an approach requires the presence of accurate estimates for the activity durations and costs of the project activities. While many studies have investigated the project management domain from different angles, they all—implicitly or explicitly—agree that good forecasting is a necessary requirement for coping with the *entropy of projects*, but this requires *energy*, which is the managerial effort of the project manager and her team.

Hence, accurate estimates should ideally be based on a mix of data for similar past projects and human judgement (the expertise often so readily available in the project team). Many of the simulation studies in the literature clearly opt for using well-known statistical distributions to model activity uncertainty, and randomly vary the parameters for the average duration and standard deviation without really knowing what realistic values are. Despite the relevance of such studies, they do not take any human judgement into account when estimating the distribution parameters, and hardly make use of data of past projects. Instead, they simply rely an arbitrarily chosen numbers for the distribution parameters without a link to real projects. The idea of calibrating data is to overcome the shortcomings of these simulation studies by relying on data of past projects to fit probability distributions, without ignoring the observation that these data are prone to human biases and possible misjudgements.

**Figure 1:**
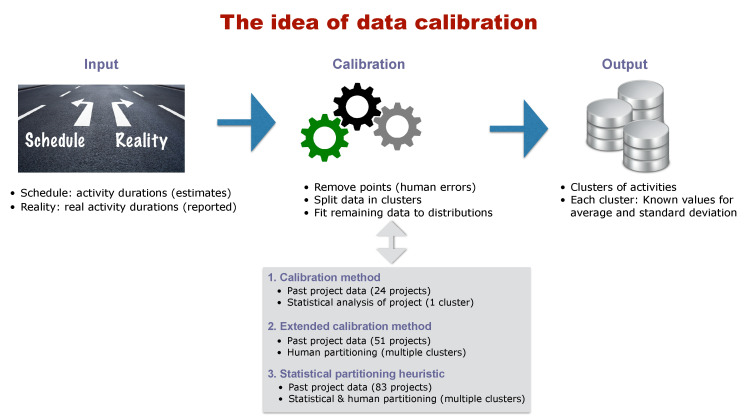
The idea of calibrating project data.

Figure 1 gives a graphical summary of the central idea of calibrating project data for activity duration distributions. A calibration method is a method to filter data of empirical projects (inputs) by removing parts (calibration) that cannot be used further in the analysis, and to identify the distribution parameters for activity duration that appears the most appropriate in a real-life context. The goal is to classify the project activities in clusters that have identical values for the parameters (average and variance) of a predefined probability distribution (outputs). The three parts (input–calibration–output) are briefly summarized along the following lines, followed by some details about the existing calibration methods.

- **Input:** The input data should exist of a set of empirical projects that are finished and for which the outcome is known. More specifically, the empirical project data should consist of a set of planned activity durations (estimates made during the schedule construction) and a set of known real activity durations (that are collected after the project is finished).
- **Calibration:** The calibration phase makes use of the input data (planned and real activity durations) and performs a sequence of hypothesis tests to split the set of activities into clusters (partitions) with similar characteristics. Throughout these hypothesis tests, it is assumed that the activity durations follow a predefined probability distribution, but a calibration method differs from an ordinary statistical test since it recognizes that the reported values in the empirical data might contain some biases. More precisely, the data might be biased due to the presence of the Parkinson effect (activities that finish early are reported to be on time (hidden earliness)) as well as rounding errors (real activity durations are rounded up or down when reported). In order to overcome these potential biases, the calibration method starts with a sequence of hypothesis tests (for which the null hypothesis is that all activity durations follow the predefined distribution), and, if the hypothesis cannot be accepted, a portion of the activities of the project has to be removed from the set to correct for the previously mentioned biases. This approach continues until the remaining set of project activities follows the predefined distribution (i.e., the test is accepted), and then the value for the average and variance of this distribution can be accurately estimated.
- **Output:** The ultimate goal of the data calibration phase is to define one or multiple clusters of activities with similar and known values for the parameters for the predefined probability distribution (i.e., average durations and standard deviation). These values can be used to better predict the project outcome, and since the activity uncertainty is then no longer set as randomly chosen values (as is often the case in simulation studies) but based on realistic values, it should enable the project manager to better predict the project outcome and reduce the project uncertainty more efficiently. Hence, calibration methods aim at better estimating the activity and project uncertainty (i) based on real project data, (ii) by taking human input biases into account, and (iii) by recognizing that not all activities should have the same values but can be clustered in smaller groups with similar values within each group, but different values between groups.

To the best of our knowledge, only two calibration methods have been proposed in literature that explicitly take the presence of the two human biases—the Parkinson’s effect and the effect of rounding errors—into account, and the current study will extend these methods to a third method. The two existing calibration procedures rely on a pre-defined distribution for the activity durations of the project, as outlined in the *calibration* step. More specifically, the *lognormal distribution* is chosen as the distribution for modelling activity duration uncertainty, which means that the null hypothesis for all calibration tests (step 2 in Figure 1) is that the division of real activity duration with the estimated activity duration from the schedule follows a lognormal distribution. While some arguments were given in previous studies why the lognormal distribution is a good candidate distribution for modelling activity duration uncertainty (see, e.g., the study by [15] who advocated the use of this distribution based on theoretical arguments and empirical evidence), this choice obviously restricts the two current and the newly presented calibration methods. Indeed, many other distributions have been used in literature to model activity duration uncertainty, such as the beta distribution (e.g., [16]), the generalised beta distribution (e.g., [17]) or the triangular distributions (e.g., [18]), but a detailed discussion on the choice of distribution and a comparison of these distributions for modelling activity uncertainty is not within the scope of our study. However, this does not mean that our study has no practical or academic value. The main goal of the calibration methods used in the study is that, although they assume that the core distribution of an activity duration is the *lognormal distribution*, it is still true that the parameters for this given distribution (such as the values for the average and standard deviation) cannot be readily seen from empirical data due to distorting human factors such as hidden earliness or rounded data. Consequently, since the calibration methods test whether activity durations follow a lognormal distribution after correcting for the Parkinson effect and rounding errors, we will refer—in line with the previous studies—to the assumed distribution for activity duration as the *Parkinson distribution with lognormal core* (PDLC).

The current paper focuses on extending the two currently existing methods to a third method, taking the weaknesses and shortcomings of the existing methods into account. A summary of the three methods is given below the *calibration* step of Figure 1. The first calibration method has been proposed by [15] and has been validated on only 24 projects by [19]. The procedure consists of a sequence of tests that removes data from the empirical database until the lognormality test is accepted for the project as a whole (no clustering). More recently, this calibration method has been extended by [20] and includes human partitioning as an initialisation step before the calibration actually starts its sequence of hypothesis tests. The underlying idea is that humans can better divide activities into clusters based on their knowledge about the project, and only afterwards, the calibration phase processes the data of each cluster to test the lognormallity of the calibration phase. A summary of both calibration methods (i.e., the original calibration method and its extension to human clustering) is given in Section 3. It is important to review both procedures since they form the foundation of the newly developed statistical partitioning heuristic discussed in the current paper. In the remainder of this paper, we will refer to the two calibrating procedures as the *calibration procedure* and the *extended calibration method*. Since both procedures contain strong similarities, they will sometimes be referred to as the two calibration procedures. The new method that will be presented in the current study—which will be referred to as a *statistical partitioning heuristic*—builds further on two currently known calibration methods in literature. The new method still relies on this basic lognormal core assumption but now extends the current calibration procedures with an automatic partitioning phase to define clusters of activities that each has the same parameters values (average and standard deviation) for their lognormal distribution. This method will be discussed in Section 4. In the computational experiment of Section 5, the three procedures will be tested on a set of 125 empirical projects (for which 83 could eventually be used for the analysis), and their performance will be compared. It will be shown that the new statistical partitioning heuristic outperforms the two other procedures but still contains some limitations that can be used as guidelines for future research.

We believe that the contribution and relevance of the current calibration study are threefold. First, and foremost, the current study presents an extended calibration method that allows the project manager to test whether clusters of activities follow a lognormal distribution for their duration. When this hypothesis is accepted, the procedure returns the values for the parameters of this distribution (average duration and standard deviation) such that they can be used for forecasting the future progress of a new project using Monte Carlo simulations. Such simulations can then be done using data from the past rather than arbitrarily chosen numbers, which is often criticised in simulation studies in the literature. Secondly, the calibration method is an extension of two previously published methods that take the same two human biases (rounding and Parkinson) into account. The extensions consist of mixing human expertise with automatic statistical testing, as well as allowing partitioning during testing rather than treating the whole project as one cluster of identical activities. Finally, to the best of our knowledge, this is the first study that calibrates data on such a large empirical dataset of 83 projects collected over several years.

Of course, our approach is only one possible approach of improving the accuracy of duration estimates, and all results should be interpreted within this limitation. Moreover, implementing such a procedure in practice requires a certain level of maturity for the project manager as it assumes that historical data are readily available. Consequently, using the new calibration method might require some additional effort as an initial investment to design a data collection methodology for past projects. Finally, even when project data are available, our approach is only beneficial if past projects are representative for future projects, which implies that some project characteristics are general and typical for the company. Consequently, in case every project is unique and totally different from the previous portfolio of projects, calibrating data would be of no use and relevance.

Of course, other studies in the academic literature have also aimed at estimating distribution parameters. However, we believe our calibration method is the first approach that does this by taking the two biases into account, and we therefore compare the new calibration method only with the two other calibration procedures using the same two biases. We believe that, thanks to the automatic nature of statistical testing in the new calibration method, our calibration method will contribute to a better forecasting of new projects, and hence to reducing the inherent uncertainty of a project with a minimum effort.

## 3. Calibration Procedures

This section gives a short summary of the two versions for calibrating data—the *calibration procedure* and the *extended calibration method*—as discussed earlier. Both calibration procedures form the foundation for the current paper, which is the reason why their main steps are repeated in Section 3.1. After this summary, the main shortcomings and areas for improvements of the extended calibration method are given in Section 3.2, and these limitations are then used to present the newly developed *statistical partitioning procedure* in Section 4.

### 3.1. Summary of Procedure

**Figure 2:**
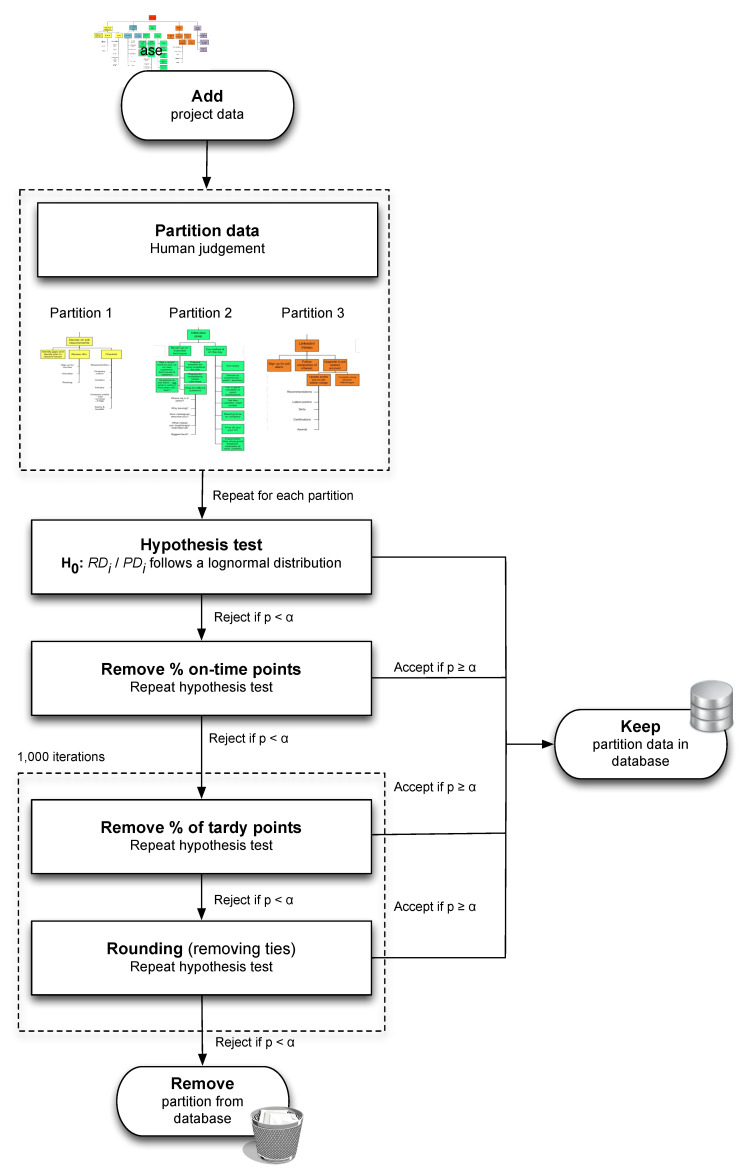
Extended calibration method.

The extended calibration method consists of five main building blocks which are graphically summarised in Figure 2. Steps S1 to S4 are identical to the four steps of the original calibration method, apart from some small technical modifications. The extended calibration method added a fifth initialisation step S0 to these four steps to cluster data into so-called human partitions. As said, these five steps (S0 to S4) are used as foundations for the new statistical partitioning heuristic discussed later, which is the reason why they are reviewed here.

Step 0 (S0). Human Partitioning

The starting point for developing the extended calibration method was inspired by the saying that “*data cannot replace human intuition*”, and that human judgement and experience of the project manager should be taken into account when evaluating data of past projects. Indeed, the original calibration method was merely a sequence of statistical tests to calibrate data, and no human input whatsoever about the project was taken into account. It is, however, well justified to state that the wonders of the human brain, although not always very reliable and subject to biases, cannot simply be replaced by a statistical data analysis, and the extension therefore mainly focused on taking this “human expertise” into account. Consequently, in order to avoid potential users of the calibration method from complaining that their human intuition would be completely ignored and replaced by a black-box statistical analysis, the gap between the dark secrets of statistical testing and the human expertise was narrowed by adding a human initialisation phase (S0) that must be executed prior to the four remaining steps of the calibration method (S1 to S4).

This initialisation phase consists of a so-called *managerial partitioning* step that splits the project data into different clusters (called partitions). The general idea is that the human expertise (the project manager’s knowledge about the project data) should come before any statistical analysis to create clusters of project data with identical characteristics. Treating these clusters separately in the remaining steps S1 to S4, rather than analysing the project data as a whole, should give the statistical calibration method more power to accept some of the project clusters, and reject others for the same project (rather than simply accepting the project data or not). Consequently, the black box analysis of the statistical calibration method is now preceded by a human input phase, and recognizes that activities of a project do not always adhere to one and the same probability distributions. Hence, the main contribution of the extension is that it assumes that computing probability distributions for activities is best done by comparing clusters of completed activities in a project rather than treating the project data as one big homogeneous dataset.

As mentioned earlier, the four remaining steps (S1 to S4) are copied from the original calibration method, only slightly extended with some minor technical adaptations to increase the acceptance rate. The only difference is that these four steps are now carried out on the different partitions separately, instead of using the project data as a whole. Each of these partitions can now pass the lognormality test (accepted partitions are assumed to contain activities with lognormal distribution, and are therefore added to the project database) or not (rejected partitions are thrown away).

In a set of computational experiments, the authors have shown that the managerial partitioning is a promising additional feature for calibrating data. Three managerial criteria have been taken into account to split the project data into partitions. More precisely, the project data were split up based on the *work packages* (WP) the activities belong to, the *risk profile* (RP) defined by the project manager as well as the estimate for the *planned duration* (PD) of each activity. The extended calibration method has been tested on 83 empirical projects taken from [21] (mainly construction projects) and results show that the additional human partitioning step increased the acceptance rate to 97% of the total created partitions.

The four remaining steps of the calibration method are now briefly summarized along the following lines.

Step 1 (S1). Hypothesis Testing (Lognormal Core)

Testing clusters (or partitions) of data using the four-phased statistical calibration method aims at creating a database of past project data (divided in clusters) in order to better understand and analyse the behaviour of new projects. For each cluster of past project data, it is assumed that the planned and real duration of its activities are known, and it is tested whether the durations of these activities follow a certain predefined probability distribution. Indeed, if the distribution of activity durations is known, its parameters can be estimated and used for analysis of a new project with similar characteristics. The hypothesis test of S1 will be repeated in each of the following steps (S2 to S4) until a final acceptance or rejection is reached. A detailed outline of the hypothesis test is given in the previously mentioned sources for the (extended) calibration method, and its main features are now briefly repeated below.

*Testing variable*: The ratio between the real duration $RD_{i}$ and the planned duration $PD_{i}$ for each activity *i* is used as the test variable in each cluster. Obviously, when $RD_{i}/PD_{i}<1$, activity *i* was completed early, $RD_{i}/PD_{i}=1$ signals on-time activities while, for $RD_{i}/PD_{i}>1$, the activity *i* suffered from a delay (these will be referred to as tardy activities).

*Hypothesis test*: The hypothesis is now that the testing variable $RD_{i}/PD_{i}$ follows a lognormal distribution for each activity *i* in the partition under study. This corresponds to testing whether $ln(RD_{i}/PD_{i})$ follows a normal distribution or not.

*Goodness-of-fit*: To assess whether the hypothesis can be accepted or not, a three-phased approach is followed. First, Pearson’s linear correlation coefficient *R* is calculated by performing a linear regression of the test variable on the corresponding Blom scores [22]. The calculated *R* value can then be compared to the values tabulated by Looney and Gulledge [23] to obtain a *p*-value. Finally, the hypothesis is accepted when p≥α with α the significance level equal to e.g., 5%. Each cluster that passes the test is added immediately to the database, while the remaining clusters will be subject to a calibration procedure.

*Calibration*: If the hypothesis is not accepted (p<α), the project data of the cluster is not immediately thrown away. Instead, the data will be calibrated, then put under the same hypothesis test again, and only then a final evaluation and decision will be made. The term *calibration* is used since it adapts/calibrates the data of a cluster by removing some of the data points. It assumes that certain data points in the cluster are subject to human biases and mistakes, and should therefore not be kept in the cluster, while the remaining points should be tested again in a similar way as explained here in S1. Two biases are taken into account, one known as the *Parkinson effect* (S2 and S3) and another to account for *rounding errors* (S4).

Steps 2/3 (S2 & S3). Parkinson’s Law

The (clusters of) project data consist of activity durations of past projects, and since the data are collected by humans, they are likely to contain mistakes. Most of the project data used in the previously mentioned studies are collected using the so-called *project card approach* of [24], which prescribes a formal method to collect data of projects in progress, exactly to avoid these human input mistakes. Nevertheless, people are and will continue to be prone to make errors when reporting numbers, and possible mistakes due to optimism bias and strategic misinterpretations will continue to exist.

For this very reason, the (extended) calibration method takes the *Parkinson effect* into account which states that work fills the allocated time. It recognizes that the reported $RD_{i}$ values are not always accurate or trustful, and they might bias the analysis and the acceptance rate of the lognormality hypothesis (S1). In order to overcome these biases, all on-time data points (S2) and a portion of the tardy data points (S3) are removed from the cluster before a new hypothesis test can be performed.

*Remove on-time points (S2)*: The procedure assumes that *all* on-time points are hidden earliness points and should therefore be removed from the cluster. More precisely, all points that are falsely reported as being completed on time, i.e., each activity with $RD_{i}/PD_{i}=1$ in a cluster that did not pass S1, are removed from the analysis. By taking this Parkinson effect into account, the cluster now only contains early and tardy points. Before a new hypothesis test can be performed, the proportion of tardy points should be brought back to the original proportion, as suggested in S3.

*Remove tardy points (S3)*: The removal of these on-time points—that actually were assumed to be early points—distort the real proportion of early versus tardy points in the data cluster, and this distortion should be corrected first. Consequently, an equal *portion* of the tardy points must be removed from the cluster too to bring the data back to the original proportion of early and tardy activities. Note that the calculation of a proportion of tardy points to remove only defines *how many* tardy activities should be removed from the cluster but does not specify which of these tardy points to remove. In the implementation of the original calibration procedure of [19], the tardy points were selected at random, while in the extended calibration method of [20], the number of tardy points were selected randomly for 1000 iterations and further analyses were carried out on these 1000 iterations to have more stable results.

After the removal of all on-time points, and a portion of the tardy points, the hypothesis test of S1 is executed again on the remaining data in the cluster, now containing a reduced amount of activities. The same goodness-of-fit criteria are applied as discussed in S1 and only when the hypothesis can not be accepted does the procedure continue with S4. Obviously, the data points of accepted clusters are added—as always—to the database.

Step 4 (S4). Coarse Time Interval

In a final phase, the remaining cluster data are corrected for possible rounding errors made by the collector of the data of the activity durations. More precisely, data points with identical values for the test variable $RD_{i}/PD_{i}$ are assumed to be mistakenly rounded up or down, as the results of the coarseness of the time scale that is used for reporting the activity durations. For example, when planned values of activity durations are expressed in weeks, it is likely that the real durations are also rounded up to weeks, even if the likelihood that the real duration was an integer number of weeks is relatively low. Therefore, corrections for rounding errors are taken into account when calculating average values of the Blom scores of these so-called tied points. More precisely, these tied points are not merged to a single score value with weight one, but rather to a set of coinciding points to retain their correct composite weight.

In the study of [20], different implementations of S4 have been tested, taking into account rounding error correction with or without including S3 and S4. It has been shown that rounding correction (S4)—although beneficial for calibrating data—is less important for accepting the hypothesis than correcting the data for the Parkinson effect, which is the reason S4 will be taken into account only after S3, as initially proposed in the original calibration procedure.

### 3.2. Limitations

Although the extended calibration procedure solved the limitations of the original calibration method while retaining its most valuable aspects, the authors still mention some limitations for their extended version, and argue that these limitations cannot be solved by minor adaptations to their procedure only. However, they also have shown that managerial partitioning (S0) adds value to the other four steps, and, hence, it would be wise not to throw away this idea. Therefore, in the current study, we propose a novel methodology that is cast into a more comprehensive and more versatile methodology called the *statistical partitioning heuristic*, which is presented in the next section. The six limitations of the extended calibration method mentioned by [20] in their Section 4.2.4 are summarized along the following lines.

- **Limitation 1.** Only on-time activities can be eliminated in S2. Moreover, if there are no on-time activities in the project, any further analysis is impossible (the proportion *x* in S3 would per definition also be zero so that no tardy points can be removed either) and no (better) fit can be obtained. Note that early activities are never eliminated from the project in the calibration procedures.
- **Limitation 2.** The *p*-value is the only measure that is applied to assess the goodness-of-fit, whereas other measures exist that could also be utilized to this end and thus prove useful to calibrate data.
- **Limitation 3.** Partitioning can only be done based on managerial criteria (using the three criteria, i.e., PD, WP and RP) and is thus influenced by human judgement.
- **Limitation 4.** The lognormality hypothesis is not tested for the tardy activities that are removed in S3. This should be done, since these activities do not follow the pure Parkinson distribution like the eliminated on-time activities in S2 do.
- **Limitation 5.** S2 only allows the elimination of *all* on-time activities, whereas removing only a fraction of them could be more optimal (i.e., better fit to the PDLC).
- **Limitation 6.** Although 1000 iterations are performed, the tardy points that are to be removed in S3 are still chosen randomly within every iteration. Deviations in results, however minor, can thus still occur.

**Figure 3:**
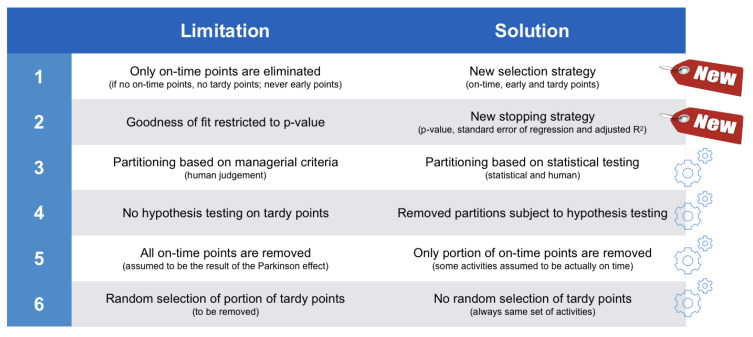
The limitations of the extended calibration procedure are used to provide solutions in the statistical partitioning heuristic.

In Section 4, the newly developed partitioning heuristic will be discussed, and it will also be shown that the discussed limitations are implicitly taken into account. A summary of the discussed limitations as well as how the new statistical partitioning heuristic has solved them are given in Figure 3.

## 4. Partitioning Heuristic

**Figure 4:**
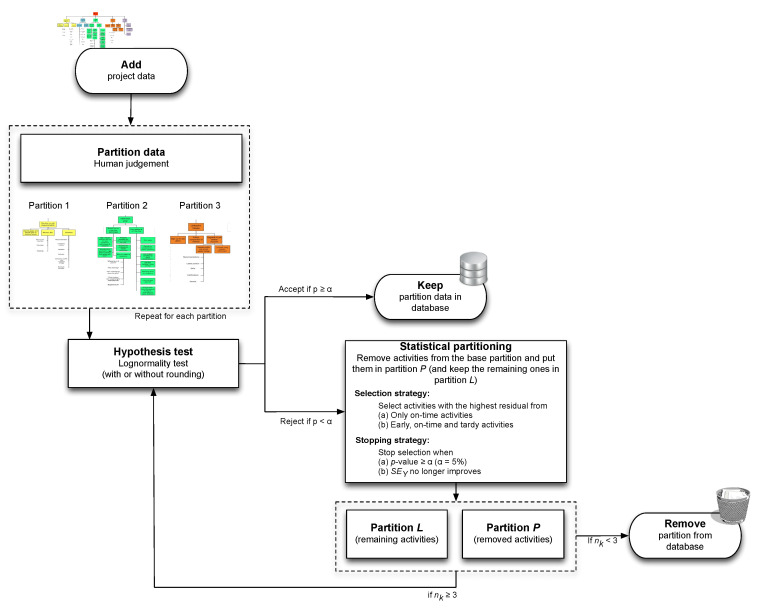
Graphical visualisation of partitioning heuristic.

The improved acceptance rate of the extended calibration method as well as its limitations have been the main driving force to develop the new statistical partitioning heuristic. It integrates the hypothesis testing approach of the original calibration method with the human partitioning philosophy of the extended calibration method, and, consequently, follows a similar methodology as both calibration procedures. The main difference is that the statistical partitioning method now partitions the project data not only based on human input, but also using a statistical methodology, and this extension has resulted in a number of significant modifications graphically summarized in Figure 4.

In this section, an overview of the newly developed partitioning method will be given subdivided in three main subsections. Each of these three sections overcome (some of) the limitations that still existed for the extended calibration procedure. We will not run through the solution approach in an explicit stepwise manner as was done in Section 3.1, but rather show where the steps (S0 to S4) have been incorporated and possibly adapted. In Section 4.3.3, the discussed limitations will be addressed chronologically and referred to when and how a particular option of the statistical partitioning heuristic solves them.

### 4.1. Human Partitioning

The procedure starts with an optional human partitioning step identical to the initialization step S0 of the extended calibration method. Since managerial partitioning has shown to be relevant for the acceptance rate of the extended calibration method, an additional non-human partitioning phase—which is the reason the new procedure is referred to as *statistical* partitioning—will be added to further split the human-based partitions into subpartitions. In the computational experiments of Section 5, results of the statistical partitioning heuristic will be reported with and without the managerial partitioning step S0.

### 4.2. Hypothesis Test

The *hypothesis test* (S1) of the statistical partitioning heuristic follows the same methodology as in both calibration procedures, and it can incorporate the data correction for rounding errors or not (S4). The test still assesses whether or not $ln(RD_{ij}/PD_{ij})$ is normally distributed by employing Blom scores and the table of Looney and Gulledge. If the correction for rounding errors (S4) is also taken into account, it still corresponds to the averaging of the Blom scores for all clusters of tied points. Therefore, it is not necessary to elaborate on each aspect of the S0 and S4 procedures in detail.

Recall that the hypothesis (S1) was also tested in steps S2 and S3 of the calibration procedures, after the removal of all on-time points and a portion of tardy points to incorporate the effect of Parkinson. As a matter of fact, the major difference between the calibration procedures and the new statistical partitioning method lies exactly in the treatment of the data for the Parkinson’s effect (S2 or S3). The (extended) calibration method aims at removing data from the project clusters to be never used again (since it follows the Parkinson effect) and only continues the hypothesis testing on the remaining portion of the data. However, the new statistical partitioning heuristic does not automatically remove data points from the clusters, but, instead, aims at splitting each partition into two separate clusters (subpartitions) and then continues testing the same hypothesis on both partitions. This iterative process of splitting and testing continues until a certain stop criterion is met, and the data of all created subpartitions that pass the test are kept in the database. More precisely, at a certain moment during the search, each subpartition will be either accepted (i.e., the data follow a lognormal distribution) or rejected (i.e., the data do not follow a lognormal distribution or the sample size of the cluster has become too small). As shown in Figure 4, we have set the minimum sample size to 3 since partitions containing too few points may get too easily accepted. The way partitions are split into two subpartitions is defined by two newly developed statistical strategies (selection and stopping), which will be discussed in the next section.

### 4.3. Statistical Partitioning

In this section, it will be shown how the statistical partitioning heuristic iteratively creates clusters of data with similar characteristics ((sub)partitions) based on statistical testing, similar to the managerial partitioning approach that aims at creating data clusters based on human input. Indeed, the statistical partitioning heuristic iteratively selects data points from a current partition and splits them into two separate clusters, and this process is repeated for each created cluster until a created subpartition can be accepted for lognormality. The specific way how these partitions are split into subpartitions does now no longer require human input but will be done using two new statistical strategies.

The so-called *selection strategy* defines which points of the current partition should be selected for removal when splitting a partition. Each removed point will then be put in a first newly created subpartition, while the remaining non-removed points are put in a second new partition, now with less points than in the original partition. This process of removing data points from the original partition continues until a certain stopping criterion is met as defined by the so-called *stopping strategy*. Once the process stops, the original partition—which we will refer to as the *base partition*—will have been split into two separate subpartitions that will both be subject to the hypothesis test again and—if still not accepted—further partitioning. In the remainder of this manuscript, the term *partition L* will be used to indicate the subpartition with the set of activities that have not been removed from the base partition, while the set of activities that were eliminated from the partition and put in a newly created subpartition is now referred to as *partition P*. It should be noted that the naming of the two partitions *P* and *L* found its roots in the testing approach of the previously discussed calibration procedures. Recall that steps S2 and S3 remove all on-time points and a portion of the tardy point from a partition. These removed points are assumed to be a subject of the Parkinson effect (hence, partition *P*) and are thus removed from the database. The remaining data points in the partition were subject to further testing for the lognormal distribution (hence, partition *L*) and—if accepted—are kept in the database. A similar logic is followed for the statistical partitioning heuristic, although the treatment of the two partitions *P* and *L* now depends on the selection and stopping strategies that will be discussed hereafter.

**Figure 5:**
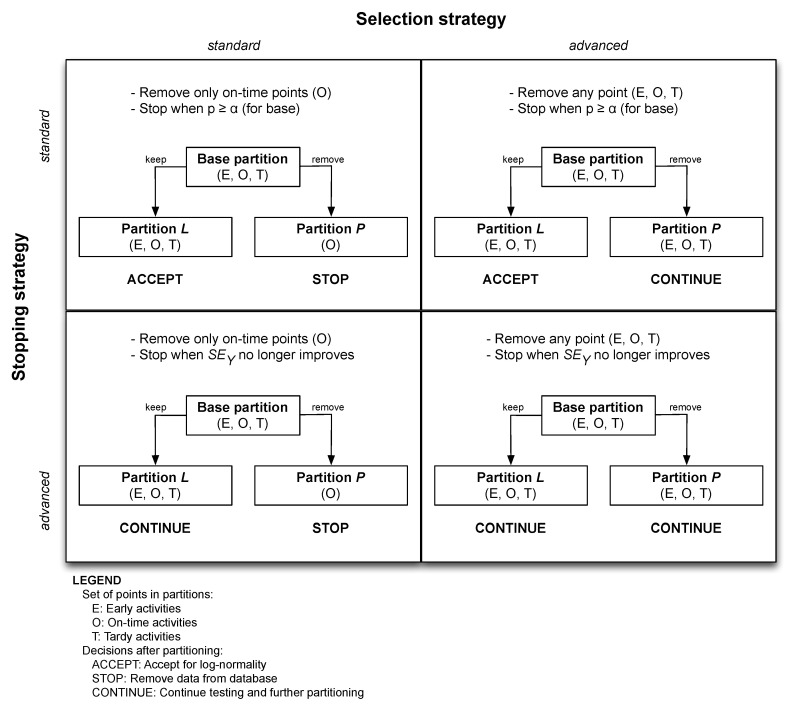
The four settings for the two strategies.

Both the selection strategy and the stopping strategy can be performed under two different settings (*standard* or *advanced*), which results in 2×2=4 different ways the statistical partitioning heuristic can be performed. Of course, these two strategies cannot work in isolation but will nevertheless be explained separately in Section 4.3.1 and Section 4.3.2. A summary is given in Figure 5.

#### 4.3.1. Selection Strategy

Recall that the partitioning heuristic splits up a partition into two new subpartitions. Partition *P* contains all the points that are removed from the base partition, while partition *L* then contains all the non-removed points (but now contains less data points compared to the base partition). The *selection strategy* defines which points will be removed from the base partition and put in partition *P*, and which points will be kept to create partition *L*, and can be done in a standard and advanced way.

The *standard selection strategy* does not differ very much from the (extended) calibration method, and defines that only on-time points can be eliminated from the base partition. As a result, partition *P* with the removed activities will then obviously exhibit a pure Parkinson distribution (since all points are on time), and no further statistical partitioning will be performed for partition *P*. Partition *L* can still consist of early, on-time and tardy points, and will be further used by the partitioning heuristic. As shown in Figure 5, no further partitioning will be performed for partition *P*, and its data are therefore thrown away (cf. STOP in Figure 5), but the specific treatment of partition *L* (ACCEPT or CONTINUE) depends on the setting of the stopping strategy, which will be discussed in Section 4.3.2.

In the *advanced selection strategy*, not only on-time, but rather *all* activities are potential candidates to be selected for removal, and thus both the resulting partitions *L* and *P* can now contain early, on-time and tardy points. This approach is called advanced since it is fundamentally different than the approach taken by the calibration procedures (S2 and S3). The most important implication of the advanced setting is that partitions in which not all activities are on time can now be created *automatically*. Indeed, the base partition will be split by eliminating activities from it, put them in partition *P* and keep the remaining activities in partition *L* until *L* attains (optimal) fit (this optimal fit will be defined by the stopping strategy discussed in the next section). The set of removed activities (partition *P*), however, can now contain both on-time and early/tardy activities (just as partition *L*) and will thus most likely not exhibit a trivial pure Parkinson distribution (as was the case for the on-time activities of partition *P* under the standard selection strategy). Therefore, this partition *P* of removed activities should also undergo a hypothesis test and possibly a partitioning phase, and so should all later partitions that are created as a result of this consecutive application of the partitioning heuristic. In that way, there is an automatic creation of partitions—hence the name statistical *partitioning* heuristic for the method—that should comprise activities that are similar to each other. Unlike the initial managerial partitioning step, no human judgement has interfered with this type of partitioning, which, from now on, we will call it for this reason *statistical* partitioning. Managerial criteria are thus no longer the sole basis for dividing activities into partitions, which addresses limitation 3 in Section 3.2. Nevertheless, managerial partitioning can of course still be performed in combination with the partitioning heuristic, just like for the calibration procedures.

While the set of activities to be removed from the base partition differs between the standard (only on-time points) and advanced (all points) selection strategy, the partitioning heuristic still needs to determine the sequence in which these activities are removed until a stopping criterion is met. Indeed, in contrast to the calibration procedures, the statistical partitioning heuristic needs to select which activity to eliminate in every partitioning step. The term *partitioning step* is used for an iteration of the partitioning heuristic in which one activity is removed. Thus, if there were 10 partitioning steps for a particular project or partition (under certain settings), then 10 activities were eliminated from that project or partition. For this purpose, the procedure calculates the residuals for all activities in the base partition. The residuals $e_{i}$ are calculated as the deviations between the empirical values $ln(RD_{i}/PD_{i})$ and the linear regression line of those values on the corresponding Blom scores. As a heuristic approach—hence the name statistical partitioning *heuristic*—the activity *i* with the biggest residual $e_{i}$ in the base partition is selected for elimination (and put in partition *P*), since it is expected that this would yield the strongest improvement in the goodness of fit (since the created partitions will be subject to a new hypothesis test again).

#### 4.3.2. Stopping Strategy

The selection strategy defines how the base partition is split into two different partitions by iteratively removing data points (activities) from it to create partitions *L* and *P*. Despite the fact that this selection mechanism controls the sequence of points to be removed using the calculation of the residuals, it does not define any stopping criterion during this iterative removal process. To that purpose, the statistical partitioning heuristic also introduces two different versions for the stopping strategy. When the stopping criteria are satisfied, the removal of activities is stopped, and the resulting partitions (*L* and *P*) are then the subject to a new partitioning iteration (i.e., they go back to S1 first before they possibly can be split further).

The *standard stopping strategy* employs the *p*-value to define the stopping criterion. More specifically, the elimination of activities stops when *p* reaches or exceeds the significance threshold α=0.05 for partition *L*. Since the *p*-value is also the condition for accepting the lognormality hypothesis in step S1, this implies that the lognormality test is automatically accepted for this partition *L*, and all its activities are assumed to follow the lognormal distribution. In this case, no further partitioning is necessary for partition *L* and all its data points are added to the database (cf. ACCEPT in Figure 5). The data points in partition *P* are treated differently, and the treatment depends on the option in the selection strategy. Indeed, since the partitioning heuristic is always applied anew to the newly created partitions, every partition *P* that is created should go back to step S1 and should be tested for lognormality if the advanced selection strategy is chosen. However, under the standard selection strategy, partition *P* only contains on-time points, and these points will obviously exhibit a pure Parkinson distribution. In this case, no further statistical partitioning will be performed and the data points are removed from the project (cf. STOP in Figure 5).

In the *advanced stopping strategy*, the statistical partitioning is no longer limited to the use of the *p*-value as the only measure for goodness-of-fit, but the activity removal halts when $SE_{Y}$ (or $R_{a}^{2}$ as a secondary stopping criterion) does no longer improve. Indeed, it applies the standard error of the regression $SE_{Y}$ as the main basis for assessing the fit, since $SE_{Y}$ is the preferred measure for this according to literature. The formula for $SE_{Y}$ is given below:

(1) $$SE_{Y}=\sqrt{\frac{\sum_{i=1}^{n}e_{i}^{2}}{n-2}}.$$

The denominator is the number of activities in the partition *n* minus 2 since there are two coefficients that need to be estimated in our case, namely the intercept and the slope of the regression line. $SE_{Y}$ is also chosen as the primary optimization criterion. By this, we mean that we deem the fit to the PDLC to be improved when the removal of the selected activity has decreased the $SE_{Y}$. Obviously, the lower the $SE_{Y}$, the better the fit. A perfect fit is obtained when all data points are on the regression line, so then all residuals are per definition zero, which, through Equation (1), implies that $SE_{Y}$ is also zero in such a case. However, in about 20% of the cases, the partitioning heuristic did not reach the optimal $SE_{y}$ when *only* that $SE_{y}$ was considered as optimization criterion; it got stuck in a local optimum. To get out of this local optimum, we added the adjusted $R^{2}$ or $R_{a}^{2}$ as a secondary stopping criterion, which—although a very straightforward approach—proved to be a highly effective solution to the problem. Indeed, after adding $R_{a}^{2}$ as a secondary optimization criterion, only 1% of the projects did not attain their optimal $SE_{y}$. For completeness, we mention the utilized formula for $R_{a}^{2}$ with respect to the standard coefficient of determination:

(2) $$R_{a}^{2}=1-\frac{n-1}{n-2}\left(1-R^{2}\right).$$

Notice that, unless $R^{2}=1$, $R_{a}^{2}$ is always smaller than $R^{2}$. In our context, we need to employ $R_{a}^{2}$ instead of $R^{2}$ to allow comparison of regression models with different numbers of observations (activities indeed get removed from the original data set). Just like for the *p*-value, the higher the $R_{a}^{2}$, the better the fit, with a maximum of 1 to reflect a perfect fit.

As mentioned before, the two settings for the stopping strategy should be used in combination with the two settings for the selection strategy, and it is important to draw the attention to the two fundamental differences with the calibration procedures. First, the treatment of the Parkinson points is fundamentally different. Recall that all on-time points are removed in the calibration procedures since they are assumed to be the result of the Parkinson effect. In the standard selection strategy, the procedure also removes on-time points, but it is no longer so that the only possibility is to remove *all* on-time points from the project. The partitioning heuristic allows the elimination of just a fraction of the on-time points in order to get a better fit (defined by the stopping strategy, i.e., *p*-value or $SE_{Y}$). The rationale behind this is that not all on-time points are necessarily the result of the Parkinson effect, as the calibration procedures implicitly assume. Some activities *are* actually on time and should thus effectively be part of partition *L*. Secondly, not only on-time points are removed, but also early and tardy points are now subject to removal. While the calibration procedures only remove a portion of tardy points to bring the number early, on-time and tardy points back to the original proportions, the statistical partitioning heuristic takes a different approach, and removes both early, and on-time as well as tardy points (under the advanced selection strategy) until the stopping criterion is satisfied. Such an approach creates partitions (*L* and *P*) that contain all kinds of activities (early, on-time and tardy) that must be subject to further partitioning, if necessary, and this is fundamentally different than the approach taken by the calibration procedures.

#### 4.3.3. Solutions

In this section, we briefly come back to the discussion of the limitations of the extended calibration procedure of Section 3.2. It will be shown that all the limitations are now solved by using a combination of the two options for the selection and stopping strategies. A summary of these solutions is also given in the right column of Figure 3.

First of all, thanks to the implementation of the selection strategy, three of the six limitations have been solved, as follows:

- **Solution 1.** The calibration procedures only removed on-time (S2) and tardy (S3) activities from the project. This is no longer true in the statistical partitioning heuristic. The advanced selection strategy states that *all* activities are selectable for removal, thus also the early and tardy ones. Early activities could never be eliminated from the project in the calibration procedures.
- **Solution 4.** The calibration procedures never apply the lognormality hypothesis to the removed tardy activities (S3). However, such a test should be performed, since these tardy activities do not follow the pure Parkinson distribution like the eliminated on-time activities in S2 do. Hence, there is no reason why these tardy points should automatically be removed from the database, and, therefore, they are subject to a new hypothesis test in the statistical partitioning heuristic.
- **Solution 6.** Thanks to the use of the $e_{i}$ criterion, 1000 iterations are no longer necessary (S3). Instead, the statistical partitioning heuristic always selects the exact same set of activities for elimination, since it now relies on the $e_{i}$ calculations. Since calculations of residuals are invariable, the created partitions would be exactly the same for every simulation run.

Secondly, the stopping strategy has been proposed in the way as described earlier to solve two other limitations:

- **Solution 2.** The *p*-value is no longer the one and only measure that is applied to assess the goodness-of-fit. Instead, the advanced stopping strategy relies on two other measures—$SE_{Y}$ and $R_{a}^{2}$—that can also be utilized to assess the goodness-of-fit.
- **Solution 5.** The Parkinson treatment of data points (S2) only allows the elimination of *all* on-time activities, whereas removing only a fraction of them could be more optimal, i.e., leading to a better fit to the PDLC.

Finally, the design of two different options (standard or advanced) for the selection and the stopping strategies is new and solves the last and most important limitation, as follows:

- **Solution 3.** The extended version of the calibration procedure added project data partitioning as a promising feature to accept lognormality, but this new feature could only be performed based on managerial criteria influenced by human judgement. The statistical partitioning heuristic has followed the same logic, but transformed it into a statistical, rather than managerial, partitioning approach. Statistical partitioning is not subject to human (mis-)judgement and not victim to human biases but does not exclude the option of human partitioning as an initialisation step (S0). In the computational experiments of Section 5, it will be shown that human and statistical partitioning lead to a higher acceptance rate of project data.

## 5. Computational Results

**Figure 6:**
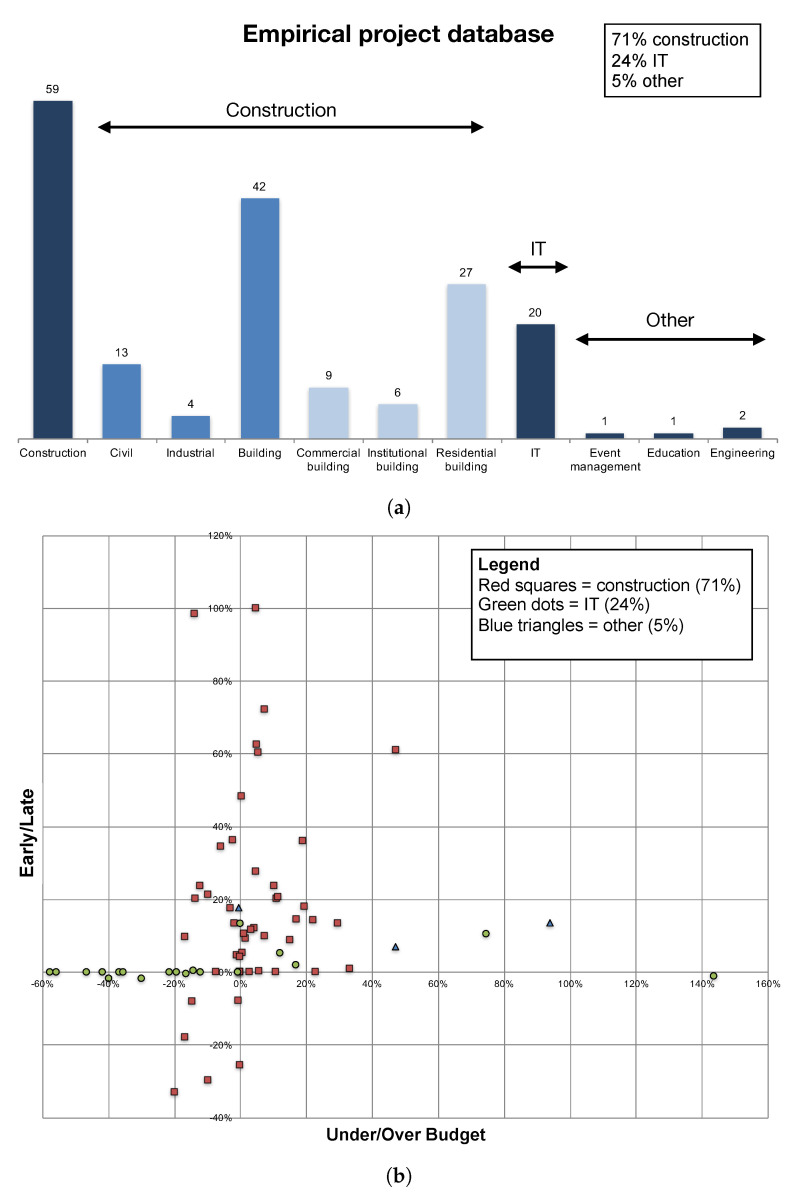
Empirical project database used for the analysis. (**a**) Sector of the 83 projects (mainly construction projects); (**b**) Project time/cost performance.

This section shows the results of a set of computational experiments on the same set of projects as used in [20]. All projects are taken from the database of [21] which consisted—at the time of introducing this database—of 51 projects. Additional projects have been added later, and has resulted in a database of 125 projects from companies in Belgium. Twenty-eight projects did not contain authentic time tracking data, and were removed from the analysis (97 left), and 14 projects only contained activities that ended exactly on time (which are assumed to be subject to the Parkinson effect). Hence, 83 remaining projects were used in the extended calibration study and will also be used in the computational experiments of the current paper. The average values for six summary statistics of these 83 projects were published in the extended calibration procedure study and are therefore not repeated here. However, Figure 6 displays a summary of the 83 projects used for the analysis. The top graph shows that more than 70% of the projects come from the construction industry, followed by almost 25% IT projects. The bottom graph displays the real time/cost performance of the projects. The graph shows that the database does not contain projects in the bottom right quadrant (over budget and ahead of schedule), but the three other quadrants contain projects with different degrees of earliness/lateness and budget underruns and overruns.

The results of our computational experiment are divided between three sections. In Section 5.1, all projects are used to test the statistical partitioning heuristic without using managerial partitioning, while Section 5.2 makes use of a subset of these projects, now also adding managerial partitioning to the tests. Finally, Section 5.3 is added with a list of limitations of the statistical partitioning heuristic that can be used as guidelines for future research in this domain.

First of all, it is very important to note that the statistical partitioning heuristic still relies on the *p*-value to determine whether or not a certain partition follows the PDLC. The reason for this is twofold. First, it allows us to compare the results of the partitioning heuristic to those of the calibration procedures—in which *p* was the only goodness-of-fit measures that was considered. In addition, second, the only other eligible measure $SE_{Y}$ does not provide a uniform basis for comparison between projects or partitions, as its numerical value strongly depends on—and can thus vary greatly with—the input values from the data set (i.e., the $ln(RD_{i}/PD_{i})$ values). In other words, no universal fit threshold can be set for $SE_{Y}$. This also explains why we will focus more on the *p*-values than on the $SE_{Y}$ results in upcoming discussions.

Secondly, it should also be stressed that $SE_{Y}$ always remains the main stopping criterion when applying the partitioning heuristic under the advanced stopping strategy. Therefore, we did not include the $R_{a}^{2}$ values in the two tables with computation results, since they are only of secondary importance. Average $SE_{Y}$ values are mentioned in the tables because of their prime role in the stopping strategy of the statistical procedure.

Finally, we consider eight different settings for the statistical partitioning heuristic, and, since each of them can be performed with or without managerial partitioning, the results had to be divided over two tables. Table 1 shows the outcomes for the application of the statistical partitioning heuristic to our database under the eight different settings without using human partitioning as an initialization step. A second table will show similar results, but now adding a human partitioning step prior to the statistical partitioning steps (Table 2). The eight settings reflect the choices that must be made for hypothesis testing (Section 4.2) and for the selection and stopping strategies of Section 4.3.1 and Section 4.3.2. Each choice can be set to either 0 or 1. To represent these different settings in Table 1 and Table 2, the code format *rounding*–*selection*–*stopping* is introduced as follows:

- The hypothesis test (S1) can be performed with (1) or without (0) rounding (S4), and will further be abbreviated as *rounding* = 0 or 1.
- The selection strategy can be set to standard (0) or advanced (1), and will be abbreviated as *selection* = 0 or 1.
- The stopping strategy can also be set to be standard (0) or advanced (1), abbreviated as mboxemphstopping = 0 or 1.

As a result, the eight settings for the parameters (*rounding*–*selection*–*stopping*) are then equal to (0-0-0), (1-0-0), (0-0-1), (1-0-1), (0-1-0),(1-1-0), (0-1-1), (1-1-1).

### 5.1. Without Managerial Partitioning

Table 1 displays the results for the statistical partitioning heuristic without managerial partitioning under the eight different settings. The table is split up in four main rows ((*a*) to (*d*)), and will be explained along the following lines.

**(*a*) # partitions:** This part displays the number of created partitions (total, average per project and maximum) as well as the percentage of projects with one up to six created partitions. All 83 available projects are considered for every setting of the partitioning heuristic. The total number of activities over these projects amounts to no less then 5068 activities (or an average of 61 activities per project), which can be deemed quite an extensive empirical dataset. Remark that the total number of partitions is equal to the number of considered projects for the settings with selection=0 (shown in the first four (-, 0, -) settings). Indeed, when only on-time points can be eliminated, partition *P* per definition follows a pure Parkinson distribution and should therefore not explicitly be considered. We thus only look at partition *L* for evaluating the partitioning heuristic with selection=0. When selection=1 (shown in the last four columns), on the other hand, the partitions created by removing any (i.e., not necessarily on-time) activity from the initial project do no longer trivially adhere to the pure Parkinson distribution. Therefore, all created partitions are considered explicitly in these cases. This explains why the number of partitions in Table 1 is bigger than 83 for settings with selection=1.

The row with the average number partitions per project (avg/p) also shows interesting results. In contrast to the situation where selection=0, there can be more (or less) than two partitions when selection is set to 1. There is a logical correspondence between the average number of partitions and the average number of partitioning steps per project (part (*b*) of the table). Indeed, the more partitioning steps that are executed, the greater the chance that an extra partition is created. As such, setting (0-1-1), which exhibited the highest number of partitioning steps for selection=1 (1705), also yields the most partitions per project, namely three on average. The minimum is observed for setting (1-1-0) (1.7 partitions per project), which also clearly showed the least partitioning steps (365). Notice that this minimum is less than 2, which means that, under this setting, there are a lot of projects for which the PDLC is accepted (i.e., p>0.05) even without elimination of a single activity, so that all activities fit the proposed distribution as a whole. This is largely due to the beneficial influence of accounting for the rounding effect through the appropriate averaging of Blom scores. When we want to optimize the fit (i.e., further decrease $SE_{Y}$), however, activities will need to be eliminated, thus producing at least one extra partition. This explains why, for the setting (1-1-1), there is on average almost one partition more per project than for setting (1-1-0) (2.6 compared to 1.7).

Furthermore, the maximum number of partitions over all projects is also displayed in Table 1, together with the grouping of the projects according to the number of partitions in which they are divided by executing the partitioning heuristic under different settings. A maximum of six partitions—which is in itself still not too much to become inconvenient to work with—only occurs for one project under setting (0-1-1). This is also the only setting for which there are more projects with three partitions than there are with two partitions, the latter clearly being the most common case and in correspondence with the situation where selection=0 (with per definition only one partition *L* and one partition *P*).

**(*b*) # partitioning steps:** When further going down the rows in the table, we see that settings with selection=1 require significantly fewer partitioning steps than settings with selection=0. This means that a potential fit can be obtained much faster by allowing all activities (i.e., early, on-time and tardy) to be removed from the base partition, which indicates a first advantage of the partitioning heuristic with respect to the calibration procedures. For setting (1-1-0), for example, an average project only needs four partitioning steps. Obviously, when the advanced stopping strategy is used (stopping=1), the number of necessary partitioning steps increases from 4 to 9. Conversely, accounting for rounding (rounding=1) appears to have a decreasing effect on the required number of partitioning steps, i.e., from 16 to 4 and from 21 to 9, which is assumed to be a positive effect given that a lower number of partitions means bigger clusters of data with similar characteristics.

**(*c*) % activities / partition:** For selection=0, we observe that partition *L* of an average project comprises between half (54%) and three quarters (73%) of the total activities, depending on the other selected options. This implies that up to about half of the activities (46%) were removed from the base partition and put in partition *P* (for setting (0-0-1)), which is quite a considerable portion provided that all these eliminated activities had to be on time. This indicates that a great part of the activities of the considered real-life projects were reported as being on time, which supports the existence of the Parkinson effect (and the rounding effect in second instance) and therefore the relevance of the applied methodologies (i.e., the calibration procedures and the partitioning heuristic to validate the PDLC). Note that no values are reported for the settings with selection=1 since, in these cases, even the partition *P* is subject to further hypothesis testing, possibly resulting in several new partitions. The division of these partitions into new partitions until the stopping criterion is met is shown by the values for the % activities in each partition under part (*a*) of this table.

**(*d*) Goodness of fit:** More importantly, one can observe that the setting (1-1-1) clearly yields the biggest *p*-value and thus the best fit to the PDLC. This *p*-value is significantly larger than that of the optimum for the extended calibration procedure when no managerial partitioning is executed (0.731>>0.385; the latter value is not shown in the table but is the maximum value of the extended calibration procedure found in Table 2 of [20]), and even larger than the overall optimum that occurs when applying initial partitioning according to RP and S4 (0.731>0.606; the latter value is the overall maximum *p*-value found in the previously mentioned study). It can thus already be stated that the statistical partitioning heuristic performs better than the extended calibration procedure, also by comparing the percentages of accepted partitions (or projects) without execution of managerial partitioning (maxima: 95%>81% for the extended calibration procedure). Moreover, accounting for the rounding effect (rounding=1) always appears to be beneficial for the validation chance of the PDLC. Similarly, there is a clear advantage of allowing every activity to be eliminated (selection=1) instead of only the on-time points (selection=0), supported by both *p*-values and accepted partitions’ percentages.

We now mention a couple of qualitative reasons why a better performance is observed for selection=1 than for selection=0. First of all, the biggest residual in a certain partitioning step will always be at least as big—and most likely bigger—in the former case than in the latter, since the algorithm can choose from *all* activities when selection=1 and not just from the on-time fraction. Eliminating an activity with a bigger residual means a stronger decrease of $SE_{Y}$ and thus a faster evolution towards the acceptance of the PDLC. This also explains why selection=1 requires fewer partitioning steps than selection=0, as mentioned earlier.

Secondly, although Table 1 did not yet consider managerial partitioning, there is statistical partitioning when setting selection to 1. This means that—in contrast to what is the case for the calibration procedures or when putting selection to zero—the early and tardy activities that show very diverse characteristics for their durations can now be assigned to different partitions for which specific distribution profiles can be defined, instead of obstinately trying to fit a single distribution profile to a set of activities that are just too heterogeneous. A good illustrative example is given by the detection of clear outliers in the project data discussed in [20] while validating their extended calibration procedure. These authors propose two straightforward criteria to select outliers, and compare their approach with the approach taken in the empirical validation of the original calibration procedure [19]. In their empirical validation of the original calibration procedure, the authors eliminated 66 activities from the set of projects as clear outliers, but they did not explicitly state how they did this. Using the two proposed criteria to detect outliers for the extended calibration procedure has resulted in the detection of the same 66 activities as being clear outliers, except for one project. This project (ID C2014-03) also had clear outliers when these two new criteria were used, but these outliers were not detected in the first empirical validation study. In the extended calibration study, it was therefore argued that failing to identify and eliminate clear outliers could lead to serious distortions in the results as a motivation for why the two criteria should always be strictly applied. This is, however, is no longer as valid as it was when the statistical partitioning heuristic was used. Using the newly proposed selection and stopping strategies, non-removed outliers would obviously exhibit the biggest residuals and thus automatically be put in a separate partition and could then no longer impede the validation of the PDLC for the other activities (and the resulting partition should be automatically removed from the project database). This also implies that it would no longer be a huge problem to not identify and eliminate the clear outliers beforehand, since the procedure would do this automatically when selection=1. The partitioning heuristic therefore becomes less prone to human error and prevents biased outcomes resulting from such errors, which of course is an advantage of the partitioning heuristic with respect to the calibration procedures and supports the applicability and robustness of the former.

### 5.2. With Managerial Partitioning

Table 2 presents more similar results than the previous table, but now with the managerial partitioning step as an initialization carried out prior to the statistical partitioning algorithm. The table no longer considers all eight settings for the statistical partitioning heuristic, but fixes the rounding value to 1 because this was shown to have a positive effect on both the partitioning efficiency (i.e., fewer partitioning steps) and, foremost, goodness-of-fit (i.e., higher *p*-value). In addition, the stopping option is also fixed to 1, since this obviously produces the better *p*-values compared to stopping=0. Moreover, the former setting in fact incorporates the latter, since, up to the point where *p* becomes greater than 0.05, both approaches run completely parallel. In contrast, the value for the selection option is not fixed, since the experiments are set up to assess its influence in combination with managerial partitioning. The settings that are included in Table 2 are thus reduced to (1-0-1) and (1-1-1). Although Table 2 (with managerial partitioning) contains more information than Table 1 (without managerial partitioning), the former will be discussed less extensively than the latter, as many aspects have already been addressed. Rather, we now focus on the most notable results and differences.

**(*a*) # Projects:** A first difference is the number of projects that are considered. This is no longer always 83 because, for some projects, the two of the three criteria for managerial partitioning were not defined by the project manager (i.e., the WPs and/or RPs of the activities were not known, cf. S0 of Section 3.1). The total number of activities that are considered is thus also less than 5068 for WP and RP as partitioning criteria, however, still adequate with a total number of activities of 3796 and 887.

**(*b*) # partitions:** The number of partitions (human) displayed in the table reflects the number of partitions that are created by performing managerial partitioning according to the different criteria. This is the initial partitioning operation (i.e., before executing the actual partitioning heuristic), and obviously yields the same partitions for both selection values. On the other hand, subpartitions are created by performing statistical partitioning and are therefore only present when selection=1. In that case, each of the partitions obtained from managerial partitioning is further divided into smaller partitions—therefore called *subpartitions*—using the statistical partitioning heuristic. This means that each project in fact goes through two consecutive partitioning phases when the partitioning heuristic is applied with setting (1-1-1) and including managerial partitioning. The number of subpartitions is obviously larger than the number of partitions, and even reaches 631 over 53 projects for the WP criterion. This comes down to almost 12 subpartitions per project, which might be a bit much to be practical and less relevant since this implies an average of only six activities per subpartition. However, this is not a problem when one of the other managerial criteria is applied, with an average of about five subpartitions per project. The main reason is that project managers apparently define way too much WPs, on average eight per project, with an excessive maximum of 26 WPs for one project. This issue could be resolved by stimulating project managers to limit the number of identified WPs through consideration of higher-level classification criteria.

**(*c*) # partitioning steps:** The number of partitioning steps do not fundamentally differ between the two tables and the table still shows that the setting with selection=1 requires significantly fewer partitioning steps than the setting with selection=0. Furthermore, the introduction of managerial partitioning does not seem to increase the average number of partitioning steps (this remains about 9 (between 8 and 10) for (1-1-1) like in Table 1), which means that the computational effort to partition the data remains just as low.

**(*d*) % activities / partition:** The percentage of activities per partition differs between the two tables. For the setting with selection=0, partition *L* on average still comprises about 80% (between 77% and 79% as shown in row ‘% act partition *L*’) of the initial activities, and even 90% for the WP criterion. This is much more than the 59% for (1-0-1) without managerial partitioning from Table 1. Hence, in order to obtain a fit to the PDLC, a far smaller portion of (on-time) activities needs to be removed from the managerial partitions than was the case for the complete project. This indicates that the application of managerial partitioning criteria is indeed relevant and beneficial, and that the definition of them by project managers should thus be stimulated.

**(*f*) Goodness of fit:** The absolute best fit so far in this research is obtained by applying the partitioning heuristic with setting (1-1-1) in combination with managerial partitioning according to the criterion that already proved most profitable in an earlier study, namely RP. The average *p*-value of 0.811 is significantly higher than the maximum for the extended calibration procedure, which is 0.606 for partitioning step S4 preceded by managerial partitioning according to—also—RP. The percentage of accepted partitions is equal and very high (97%) for both, so we can conclude that the partitioning heuristic outperforms the calibration procedures, regardless even of its qualitative benefits concerning flexibility and robustness. Therefore, we will no longer consider the (extended) calibration procedure in the rest of the discussion.

However, the mentioned *p*-value of 0.811 is not exceedingly higher than that for partitioning setting (1-1-1) combined with either of the other managerial criteria (*p* ranging from 0.756 to 0.783) or even without managerial partitioning (p=0.731; see Table 1), and also the partitioning setting (1-0-1) combined with managerial partitioning according to—again—RP comes close with a *p* of 0.741. The reason for this is that a combination of managerial partitioning and statistical partitioning (which occurs when selection=1) should in fact be seen as a ‘double’ optimization. Both partitioning approaches already perform very well separately, but combining them takes the distribution fitting another (small) step closer to ‘optimal’ partitioning. Furthermore, managerial and statistical partitioning do not only perform well on their own; they are mutually also quite comparable. To show this, we need to compare the partitioning heuristic with setting (1-1-1) (so without advanced statistical partitioning) and no managerial partitioning (see Table 1) and that with setting (1-0-1) (so without advanced statistical partitioning) and managerial partitioning according to RP (see Table 2). Remarkably, both exhibit almost identical *p*-values (0.731 versus 0.741) and accepted partitions percentages (94% versus 95%). This observation is in fact hugely promising, as it indicates that we can just perform the partitioning heuristic with inclusion of the statistical partitioning (i.e., set selection to 1) and still obtain very relevant partitions without requiring realistic input for managerial criteria (i.e., WPs or—even better—RPs accurately defined by the project manager). Statistical partitioning is no longer—or at least far less—prone to human judgement and bias than managerial partitioning. In the latter case, project managers indeed need to *accurately* define the WPs or RPs, otherwise the resulting partitions would be faulty and unrealistic anyhow. It might be beneficial to bypass this uncertain human factor, and thus create a more solid and trustworthy methodology for categorizing activities into risk classes and assigning specific distribution profiles to them. The partitioning heuristic developed in this section allows just this. Apart from the discussion of either managerial or statistical partitioning (or both) being preferred, our results clearly show that it is essential to create partitions for a project in order to obtain decent fits of the activity durations to the PDLC.

### 5.3. Limitations

Notwithstanding the substantial improvements of the statistical partitioning heuristic with respect to the calibration procedures, some extensions to the procedure itself and to the related research could still be made in the future. We now present a few limitations of the current research and propose several potential advances that could be made in these areas.

1. The statistical partitioning heuristic—as the name itself indicates—is still a heuristic and therefore produces good but not (always) optimal results. Indeed, removing the activities with the biggest residuals $e_{i}$ as long as the $SE_{Y}$ of the considered base partition (put in partition *L*) decreases is a plausible and logical approach. However, it is not optimal for multiple reasons. First, it is no certainty that the biggest residual always designates the best activity to eliminate (i.e., which produces the biggest decrease of $SE_{Y}$). Second, it is not assessed what the future impact (i.e., over multiple partitioning steps) of this removal would be on the remaining activities in partition *L* (e.g., maybe it would be more optimal to remove two other high-residual activities instead of that with the biggest residual, but the algorithm does not analyse this). In addition, third, when removing an activity from partition *L*, it becomes part of another partition (i.e., partition *P*), but we do not check the influence of this operation on partition *P* (for all we know, it could deteriorate the $SE_{Y}$ there). In addition, then there still is the issue of $SE_{Y}$ being susceptible to lapse into a local optimum, which we now—also heuristically—addressed by considering $R_{a}^{2}$ as a secondary optimization criterion. The ultimate goal would be to develop an algorithm that divides the activities of a project into partitions that all pass the lognormality test (with possible exception of some clear outliers), and moreover, show an average $SE_{Y}$ over all partitions that is as low as possible (or a *p*-value that is as high as possible). The advanced algorithm could, for example, contain a fine-tuning stage in which activities can be shifted from one partition to another in order to further improve the overall $SE_{Y}$ or *p*. Furthermore, a limit could be set for the minimal allowed partition size, to make the partitions themselves more meaningful and comparison with partitions from similar future projects more workable. We have now set the minimum size of each partition arbitrarily to 3.
2. The employed project database is large for an empirical data set, but still rather limited in comparison to simulation studies using artificial project data. Therefore, the database should ever be further expanded, so that future empirical studies based on it can keep increasing their validity and generalizability.
3. Currently, we only considered the initial partitioning according to one managerial criterion at a time. This could be extended to the application of multiple consecutive criteria. For example, the PD criterion could be performed after the project has already been partitioned according to RP. In that way, we get even more specific partitions that should exhibit activities that are more strongly related. Furthermore, the extra managerial partitioning could be applied together with or instead of the statistical partitioning (i.e., if selection=1).
4. Furthermore, the managerial partitioning criteria should not stay limited to PD, WP and RP. These are perhaps some of the most obvious and logical criteria, but there can still be others that might show even greater distinctive power for dividing a project into adequate partitions. These extra managerial partitioning criteria could be harvested from more empirical research, for example, into the drivers of project success. If those drivers could be reliably identified for a particular kind of project, they could also provide a good basis for grouping similar activities that thus show similar risks (and should therefore belong to the same partition).

## 6. Conclusions

Studies have shown that, just like any physical system, projects have entropy that must be managed by spending energy, and this process of energy is called project management. In order to manage the project uncertainty, accurate estimates for activity duration are crucial in order to make informed decisions. This paper presents a new statistical method to better estimate the average and variability of the activity duration distributions in order to help project manager to better manage the project uncertainty (entropy) with the lowest possible effort (energy).

The new statistical calibration method extends two existing calibration methods using an automatic partitioning heuristic. The main objective of such an extension is to improve the ability to define distribution profiles for a project’s activity duration that represent as accurately as possible the stochastic nature of the activities. The underlying assumption is that the lognormal distribution is the most appropriate distribution for modelling activity durations, but the parameters for this distribution cannot be easily extracted from empirical data due to hidden earliness and rounded values for the reported activity durations. These procedures were utilized as a starting point for developing a much more extensive calibration procedure, which has programmed in C++ and empirically validated on the dataset consisting of more than 5000 activities. These input data come from the real-life project database created by [21] and is freely available at www.or-as.be/research/database.

The previous calibration methods have shown promising results, but also some limitations, and these are also discussed in the current study. First, the original calibration procedure of [19] did not allow the project to be divided into partitions of activities that intrinsically adhere to the same distribution profile. For this reason, [20] have proposed an extended calibration method by introducing the ability of managerial partitioning using human input such as planned duration, the structure of the work breakdown structure or the risk profiles defined for each activity. This extended calibration method proved extremely favourable and confirmed that partitioning is a promising direction for proving the realism of the lognormal distribution for activity duration. Despite this improvement, managerial partitioning is based on criteria defined by the project manager, and, as the project manager is a human being, these criteria are susceptible to bias in human judgement.

To bypass this problem, we developed a completely new approach in the current study which we called the *statistical partitioning heuristic*. It is foremost a *statistical* procedure in contrast to the managerial procedure that requires human input. Moreover, the *partitioning* approach, which was shown to be promising in the extended calibration study, is kept as a *heuristic* tool (i.e., there are other ways of doing the partitioning) in the best possible—but not necessarily optimal—way. Consequently, in statistical partitioning, well-chosen activities that do not fit within a certain partition are eliminated from that partition and assigned to another, which is then also adapted until a fit is reached. The results obtained from this are very good, and almost perfectly match those from performing managerial partitioning in the extended calibration method.

This observation is certainly advantageous, as it suggests that equally adequate partitions can be obtained through the proposed statistical procedure without being susceptible to human bias or, moreover, requiring the definition of managerial criteria. Since project managers are now always able, or willing, to define values for the managerial criteria for all activities, an automatic procedure can replace their cumbersome task. It is therefore advised to perform the statistical partitioning heuristic with the incorporation of advanced selection and stopping strategies for receiving the most appropriate and trustworthy distribution profiles for the activity durations. However, when it is certain that the managerial criteria have been properly defined, managerial partitioning can be executed in combination with (in fact, prior to) the statistical partitioning. Despite the promising results in this study, future research topics can be derived from Section 5.3, since addressing the limitations of the current automatic partitioning heuristic could indeed further advance our research.

## Figures and Tables

**Table 1:** Results for the partitioning heuristic without managerial partitioning.

		Partitioning Setting							
		(Rounding–Selection–Stopping)							
		(0-0-0)	(1-0-0)	(0-0-1)	(1-0-1)	(0-1-0)	(1-1-0)	(0-1-1)	(1-1-1)
(*a*)	# partitions (total)	83	83	83	83	195	145	249	215
	# partitions (avg/p)	-	-	-	-	2.3	1.7	3.0	2.6
	# partitions (max)	-	-	-	-	5	3	6	5
	1 partition [%]	-	-	-	-	13	36	4	6
	2 partitions [%]	-	-	-	-	51	53	25	42
	3 partitions [%]	-	-	-	-	25	11	47	40
	4 partitions [%]	-	-	-	-	10	0	17	11
	5 partitions [%]	-	-	-	-	1	0	6	1
	6 partitions [%]	-	-	-	-	0	0	1	0
(*b*)	# partitioning steps	2566	2177	2771	2634	1361	365	1705	771
	/project	31	26	33	32	16	4	21	9
(*c*)	% act / partition *L*	62	73	54	59	-	-	-	-
	% act / partition *P*	38	27	46	41	-	-	-	-
(*d*)	avg. $SE_{Y}$	0.271	0.229	0.250	0.212	0.257	0.191	0.264	0.139
	avg. *p*	0.075	0.193	0.280	0.479	0.219	0.362	0.461	0.731
	accepted partitions [%]	61	72	61	72	90	95	86	94

**Table 2:** Results for the partitioning heuristic with managerial partitioning.

		Partitioning Setting							
		(Rounding–Selection–Stopping)							
		(1-0-1)				(1-1-1)			
		PD (x4)	PD (x5)	WP	RP	PD (x4)	PD (x5)	WP	RP
(*a*)	# projects	83	83	53	21	83	83	53	21
	avg. # activities	61	61	72	42	61	61	72	42
	tot. # activities	5068	5068	3796	887	5068	5068	3796	887
($b_{1}$)	# partitions (human)	232	213	426	65	232	213	426	65
	# partitions (avg/p)	2.8	2.6	8.0	3.1	2.8	2.6	8.0	3.1
	# partitions (max)	4	4	26 *	6	4	4	26 *	6
	1 partition [%]	4	6	36	0	4	6	36	0
	2 partitions [%]	32	40	45	24	32	40	45	24
	3 partitions [%]	45	46	8	52	45	46	8	52
	4 partitions [%]	19	8	7	19	19	8	7	19
	5 partitions [%]	0	0	2	0	0	0	2	0
	6 partitions [%]	0	0	2	5	0	0	2	5
($b_{2}$)	# subpartitions (statistical)	-	-	-	-	423	399	631	117
	# subpartitions (avg/p)	-	-	-	-	5.1	4.8	11.9	5.6
	# subpartitions (max)	-	-	-	-	4	4	5	4
	1 subpartition [%]	-	-	-	-	40	37	59	34
	2 subpartitions [%]	-	-	-	-	40	41	35	54
	3 subpartitions [%]	-	-	-	-	18	19	4	11
	4 subpartitions [%]	-	-	-	-	2	3	1	1
	5 subpartitions [%]	-	-	-	-	0	0	1	0
(*c*)	tot. # partitioning steps	2150	2246	835	348	689	751	555	182
	/project	26	27	16	17	8	9	10	9
(*d*)	% act. partition *L*	79	78	90	77	-	-	-	-
	% act. partition *P*	21	22	10	23	-	-	-	-
(*f*)	avg. $SE_{Y}$	0.161	0.171	0.196	0.101	0.108	0.130	0.146	0.088
	avg. *p*	0.614	0.589	0.658	0.741	0.774	0.756	0.783	0.811
	accepted (sub)partitions [%]	88	85	92	95	97	94	97	97

* For partitioning criterion WP, a different scale applies for the next six rows: 1 / 2 / 3 / 4 / 5 / 6 partition(s) should be regarded as 1-5/6-10/11-15/16-20/21-25/26-30 partitions, respectively.
